# Observer ratings of neighborhoods: comparison of two methods

**DOI:** 10.1186/1471-2458-13-1024

**Published:** 2013-10-29

**Authors:** Elena M Andresen, Theodore K Malmstrom, Mario Schootman, Fredric D Wolinsky, J Philip Miller, Douglas K Miller

**Affiliations:** 1 Institute on Development & Disability, Oregon Health & Science University, Portland, OR, USA; 2 Department of Neurology & Psychiatry, School of Medicine, Saint Louis University, 1438 S. Grand, St. Louis, MO 63104, USA; 3 Departments of Medicine and Pediatrics, Washington University School of Medicine, 4444 Forest Park Parkway, Box 8504, St. Louis, MO 63108, USA; 4 Departments of Health Management and Policy, Internal Medicine, and Adult Nursing, the University of Iowa, N211 CPHB, 105 River St., Iowa City, IA 52242, USA; 5 Division of Biostatistics, Washington University School of Medicine, 660 South Euclid Avenue, Campus Box 8067, St. Louis, MO 63110, USA; 6 Regenstrief Institute, Inc., and Center for Aging Research, Indiana University School of Medicine, 410 West 10th Street, Suite 2000, Indianapolis, IN 46202-3012, USA

## Abstract

**Background:**

Although neighborhood characteristics have important relationships with health outcomes, direct observation involves imperfect measurement. The African American Health (AAH) study included two observer neighborhood rating systems (5-item Krause and 18-item AAH Neighborhood Assessment Scale [NAS]), initially fielded at two different waves. Good measurement characteristics were previously shown for both, but there was more rater variability than desired. In 2010 both measures were re-fielded together, with enhanced training and field methods implemented to decrease rater variability while maintaining psychometric properties.

**Methods:**

AAH included a poor inner city and more heterogeneous suburban areas. Four interviewers rated 483 blocks, with 120 randomly-selected blocks rated by two interviewers. We conducted confirmatory factor analysis of scales and tested the Krause (5-20 points), AAH 18-item NAS (0-28 points), and a previous 7-item and new 5-item versions of the NAS (0-17 points, 0-11 points). Retest reliability for items (kappa) and scales (Intraclass Correlation Coefficient [ICC]) were calculated overall and among pre-specified subgroups. Linear regression assessed interviewer effects on total scale scores and assessed concurrent validity on lung and lower body functions. Mismeasurement effects on self-rated health were also assessed.

**Results:**

Scale scores were better in the suburbs than in the inner city. ICC was poor for the Krause scale (ICC=0.19), but improved if the retests occurred within 10 days (ICC=0.49). The 7- and 5-item NAS scales had better ICCs (0.56 and 0.62, respectively), and were higher (0.71 and 0.73) within 10 days. Rater variability for the Kraus and 5- and 7-item NAS scales was 1-3 points (compared to the supervising rater). Concurrent validity was modest, with residents living in worse neighborhood conditions having worse function. Unadjusted estimates were biased towards the null compared with measurement-error corrected estimates.

**Conclusions:**

Enhanced field protocols and rater training did not improve measurement quality. Specifically, retest reliability and interviewer variability remained problematic. Measurement error partially reduced, but did not eliminate concurrent validity, suggesting there are robust associations between neighborhood characteristics and health outcomes. We conclude that the 5-item AAH NAS has sufficient reliability and validity for further use. Additional research on the measurement properties of environmental rating methods is encouraged.

## Background

Characteristics of local neighborhoods are now frequently incorporated into research assessing factors associated with health behaviors and outcomes [1-6]. Empirical studies arise from a number of theoretical frameworks, including an overarching public health socio-ecological framework [7] and more finely nuanced theories and conceptual frameworks regarding specific neighborhood characteristics and hypothesized outcomes such as walking and physical activity [8,9], obesity [10], disability and physical function [11-16], parenting [6], and specific health conditions such as depression [17,18], diabetes [19], and inflammatory markers [20]. Observer-rated measures for research on the effects of neighborhoods include a range of options related to research objectives, hypotheses, and theoretical models. One example is social disorganization theory, which provides an organizing framework for understanding neighborhood effects on depression [12,18,21]. Among published measurement instruments, there are some reports of measurement qualities (e.g. [22-26]). However a full treatment of issues like reliability, validity, and psychometric evidence of scale performance is lacking for most measures [25,27].

In nine years of tracking the African American Health (AAH) Cohort, we fielded two neighborhood observer rating systems, initially at two different waves. Both rating systems use global approaches to neighborhood effects theory and analyses of diverse health outcomes. One was an existing brief five-item measure [28] which had some good measurement properties [22]; however, we found that it had excessive rater variability. The second was the Neighborhood Assessment Scale [NAS] specifically adapted for use in the AAH [29,30] and consisting of 18 items. Based on the potential utility in some field studies for a shorter rating scale, we conducted further analysis among these items, resulting in a seven-item version that represented an improvement over the Krause five-item scale but still contained rater variability [31]. In the present study, we re-fielded both measures in 2010 hypothesizing that enhanced training and field methods would decrease rater variability. Further, this head-to-head comparison allowed us to examine if the broader psychometric characteristics of the measures under refined field methods were similar between measures, including confirmatory factor analysis.

## Methods

### Sampling and rater assignments

The baseline sampling strategy for the parent AAH study involved two geographic areas that differ widely in socioeconomic status (SES) [32-34]. One catchment area is a poor, predominantly African American inner city neighborhood where 24% of AAH respondents reported annual incomes under $10,000. The second catchment area is a suburban, integrated neighborhood with variable individual and neighborhood economic status, where only 8% of AAH respondents reported annual incomes under $10,000 during our baseline interviews in 2000 [32,34]. Households were sampled based on a multi-stage cluster design. We identified block groups with at least 10% African Americans in the 1990 census using geographic information system data. We then randomly selected first area segments within block groups and then housing units within each selected segment. If the housing unit contained two or more eligible persons (based on age and race criteria), interviewers used Kish tables [35] to select one of them for possible participation. At Wave 10 (spring-summer of 2010), 582 respondents were interviewed, and 569 of these still lived in (n=385, 67.7%) or near enough to (n=184, 32.3%) one of the two original catchment areas to have their current neighborhood assessed. Because in 15.1 percent of blocks there were 2 or more AAH addresses, a total of 483 blocks were rated by interviewers. Household interviews occurred during the preceding weeks (mean 10±3 weeks) before the neighborhood rating phase that is the focus of this paper.

The addresses of participants were standardized using ZP4 [36], which is the official United States Postal Service® data file that provides a tool for automatically determining the correct mailing address, ZIP + 4® code, and mail carrier route number for any location in the U.S. Next, we geocoded addresses using the 2009 U.S. Nationwide Streets StreetMap™ [37] under ArcGIS® ArcMap™ 9.3.1 [38]. Of eleven participant addresses which could not be matched in ArcGIS® [38], five were geocoded with the Tele Atlas EZ-Locate web geocoder [39], and the remaining six were found using Google Earth [40]. The name and house number range for the side of the street, odd or even, for each segment was verified to contain the participant address to avoid chance association only on proximity. A series of grayscale maps with the assigned street segments were generated to facilitate geographic grouping of rating areas and reduce travel time for raters. A total of 120 segments were randomly selected and rated by two raters and thus could be used in inter-rater assessments of items and scale scores.

### Training and quality control

We selected 4 of 11 field interviewers for this neighborhood rating phase based on their experience with AAH, the field supervisor’s rating of their quality, and the large number of interviews that they conducted relative to others in the prior participant interview phase. Raters received a total of six hours of “classroom” training as a group, and three hours of field training during two sessions in the field. Briefly, the “classroom” training consisted of an initial four-hour session that included a presentation and discussion of the paper and pencil rating forms, review of the Question-by-Question training guide, followed by photo examples of our St. Louis neighborhoods with specific ratings provided. For example, digital photos with light versus heavy litter were displayed and discussed. Raters then practiced using case example photos, where all visible attributes from both the Krause and AAH NAS forms were listed in a practice response book for independent rating. We then reviewed and discussed ratings of the cases, focusing on consistency with the answer sheet.

Following the classroom session, the rater team and trainer spent two hours walking in a neighborhood outside the two study catchment areas with heterogeneous street segments, reviewing and rating each block face we visited and discussing ratings for consistency. Each rater then independently visited and rated several sample blocks outside the catchment areas. In the second day of “classroom” training, we reviewed and discussed questions and issues about their experiences during an additional two hours. Finally, one investigator attended field ratings for three assigned street segments for each of the four raters, providing independent ratings with rater debriefing immediately after the rating.

Two investigators reviewed the first 51 forms completed by the four raters for quality assurance (skipped or incomplete items, unusual or inconsistent patterns between similar items of the Krause and AAH forms), and debriefed these with raters. Consistency of ratings and quality control of the completed ratings were the foci at each of these phases of training and initial field data collection phases. During the formal data collection phase of block segments for the AAH participants, all forms were submitted weekly and reviewed by the field supervisor or one of the investigators. Forms with missing or inconsistent information, although uncommon, were returned immediately to raters for correction, occasionally requiring that they revisit a block for a missing item.

### Data from participant interviews

Several measures in the present study were obtained from data collected in the field interview phase of AAH that occurred in the months before the neighborhood rating phase. In-person interviewer training for this phase was conducted during a full week, similar to each of our two prior in-persons interview waves, including training by a member of the investigator team who is a clinician [41]. Lower body functional limitations (LBFL) was measured as a summary of five self-reported items from the Nagi physical performance scale (0 = no difficulties, 1 = difficulty), which were summed to form the outcome measure (ranging from 0 to 5) in the present study [42]. Items included difficulties in walking a quarter of a mile; walking up and down 10 steps without rest; standing for 2 hours; stooping, crouching, or kneeling; and lifting 10 pounds [43].

The Short Physical Performance Battery (SPPB) summary score is a test comprised of three lower body measures: a hierarchical test of standing balance; five consecutive chair rises; and usual gait speed [40,43]. Interviewers instructed participants in proper technique and then recorded the performance for each component using a standardized protocol. We constructed the component scores of 0–4 based on cut-points previously validated in our cohort, resulting in total scores that could range from 0 to 12 points with higher scores representing better function [40]. The Peak Expiratory Flow (PEF) was measured using a standard flow meter (Assess Flow Meter by Respironics, Cedar Grove, NJ) with the participant standing for the assessment. Their performance was recorded as the average of the maximum liters/minute over three trials [44]. Participants also were asked to report their current health status on a five–point Likert scale as excellent, very good, good, fair, or poor.

### Rating scales

The Krause scale [28] rates the condition of the street where each respondent lived on five characteristics by observing both sides of the block (houses/buildings, noise, air quality, streets, yards/sidewalks). Raters assigned a category for each item using the following scale: 1=excellent, 2=good, 3=fair and 4=poor (see Table 1). The five items combine for a total score of 5 (best) to 20 (worst) conditions [28].

**Table 1:** Comparisons of interviewer rating of the Krause, and the African American Health Neighborhood Assessment Scale (AAH NAS) items and scales

**Krause five-item scale**					
**Items and scale**	**Categories/Range**	**Retest pairs n=120**		**Catchment area comparison #**	
		Kappa+/ICC*	**Adjusted Kappa+**	**Inner city**	**Suburbs**
				**n=237**	**n=332**
1. Condition of houses, buildings	All 5 items coded 1–4; Excellent, Good, Fair, Poor	0.32	NA	2.0 ± 0.8	1.5 ±0.6
2. Amount of noise		0.26	NA	1.8 ± 0.9	1.6 ±0.8
3. Air quality		−0.17	NA	1.3 ± 0.5	1.2 ± 0.5
4. Condition of street		0.03	NA	1.5 ± 0.7	1.6 ± 0.7
5. Condition of yards, sidewalks		0.32	NA	2.1 ± 0.9	1.6 ±0.7
Total Rating Score (Items 1–5) Ѱ	Range 5–20 (observed range 5–16). 23.1% of scores were the minimum value of 5.	0.19	NA	8.6 ± 2.6	7.5 ± 2.4
**African American Health Neighborhood Assessment Items and Scales**					
**Items and scales**	**Categories/Range**	**Retest pairs n=120**		**Catchment area comparison**	
*Items that do not contribute to scores shown in italics*					
		Kappa+/ICC*	**Adjusted Kappa+**	**Inner city**	**Suburbs**
**Items rating entire street**				**n=237**	**n=332**
1. Volume of traffic	None, light, moderate, heavy (0–3)	0.38	NA	0.96 ± 0.93	0.73 ± 0.79
2. Condition of street	Very good, moderate, fair, poor (0–3)	0.09	NA	2.43 ± 0.72	2.37 ± 0.75
3. Amount of noise	Very, fairly quiet; somewhat, very noisy (0–3)	0.24	NA	0.84 ± 0.81	0.53 ± 0.68
4. Smells	None, any (0,1)	---	0.97	1.3%	3.3%
5. Dirt or dust	None, any (0,1)	---	1.00	0.8%	0.6%
**Items rated on block face of respondent’s residence**					
6. Abandoned car	None, any (0,1)	−0.03	0.88	5.1%	2.7%
7. Beer, liquor bottles	None, any (0,1)	0.12	0.48	32.9%	10.6%
8. Cigarette, tobacco litter	None, any (0,1)	0.12	0.13	74.3%	45.8%
9. Garbage, litter, broken glass	None, light, moderate heavy (0–3)	0.33	NA	0.92 ± 0.82	0.48 ±0.64
*10. Land use residential*	*None, any (0,1)*	---		*97.9%*	*98.5%*
*10.a. Type (most)*	*Detached single family*	*0.58*	0.97	*53.6%*	*95.3%*
	*Private multi family*			*25.0%*	*0.0%*
	*Private apartments/townhouses*			*16.1%*	*4.7%*
	*Public housing*			*5.4%*	*0.0%*
10.b. Condition	Very well kept/good, moderately well kept, fair, poor/badly deteriorated (0–3)	0.19	NA	0.88 ± 0.82	0.50 ± 0.68
10.c. Bars/grates on doors or windows	None, any (0,1)	0.43	0.43	68.2%	35.8%
*11. Land use commercial/ business/professional/industry*	*None, any (0,1)*	*0.31*	0.82	*12.7%*	*4.2%*
*11.a. Condition*	*Very well kept/good/moderate; Fair/poor/deteriorated*	−*0.45*	−0.57	*78.8%*	*100%*
*11.b. Security blinds, iron gates*	*(0,1) None, any (0,1)*	*0.40*	−0.33	*48.5%*	*21.4%*
*12. Land use institutions (schools, churches etc.)*	*None, any (0,1)*	*0.11*	0.82	*8.4%*	*2.7%*
*13. Land use parks*	*None, any (0,1)*	---	0.97	*0.4%*	*0.3%*
*14. Land use playgrounds*	*None, any (0,1)*	*−0.01*	0.95	*0.8%*	*0.6%*
*15. Land use other recreational*	*None, any (0,1)*	*---*	0.98	*1.3%*	*0.3%*
*Summary of 13-15*	*0-3*	*−0.02*	NA	*0.03 ± 0.18*	*0.01 ± 0.13*
16. Condition of 13-15	Very well kept/good, moderately well kept, fair, poor/badly deteriorated (0–3)	−0.01	NA	3.97 ± 0.33	3.96 ± 0.33
17. Graffiti	None, any (0,1)	−0.02	0.92	5.5%	0.3%
*18. Neighborhood/Crime watch*	*None, any (0,1)*	*0.01*	0.66	*9.7%*	*10.9%*
*19. Security warning signs*	*None, any (0,1)*	*0.44*	0.61	*69.2%*	*81.9%*
20. Tobacco advertisements	None, any (0,1)	−0.01	0.93	2.5%	0.9 %
21. Alcohol advertisements	None, any (0,1)	−0.01	0.97	3.0%	1.5%
22. Home “for sale”	None, any (0,1)	0.15	0.62	13.5%	16.0%
Total 18-item Score (1–9, 10b, 10c, 13–15,17, 20–22) Ѱ	0-28 (observed range 3–20)	0.54	NA	9.2 ± 3.4	7.0 ± 3.2
Short 7-item Scale (1–3, 7–9, 10b) Ѱ	0-17 (observed range 0–13). 11.5% of scores were the minimum value of 0.	0.56	NA	5.2 ± 3.0	3.4 ± 2.8
Short 5-item Scale (3, 7–9, 10b) Ѱ	0-11 (observed range 0–9) 19.7% were the minimum value of 0	0.62	NA	3.7 ± 2.2	2.1 ± 2.0

*Intraclass correlation coefficient. ^+^ Kappa or Weighted Kappa (К) for ranked categories; adjusted kappa (Lantz & Nebenzahl, 1996). Values with --- notation could not be calculated because one or more cells of the kappa table had zero observations.

^#^Higher scores represent lower neighborhood condition quality. Proportions and 95% confidence intervals are reported for dichotomous responses, and means and standard deviations are reported for items with three or more categories, and for scales.

Ѱ Mean values between catchment areas are significantly different by t-tests at p<0.05.

The AAH NAS includes 27 items. Nine are descriptors that do not contribute to scores, and 18 items are scored for use in summary scales (see Table 1 for items and response categories). Categories of ratings for the items used for score scales were assigned larger numbers for decreasing quality categories so that higher scores represented worse neighborhood conditions. Five items (traffic volume, street condition, noise, smells, dirt/dust) are rated for the overall street environment, and the 13 remaining items ask raters to view and gauge the block face that corresponds to the subject’s residence. Some items have a set of ranked quality categories (e.g., traffic volume: none, light, moderate, and heavy), while other items are rated as yes/no (condition present or not). Table 1 provides a summary of questions and categories of the AAH NAS items. A total 18-item AAH NAS score provided a possible range of 0–28 points. Seven items were previously combined into a brief NAS that ranged from 0 to 17 points for items assessing traffic volume, condition of the street, noise, alcohol litter, tobacco litter, overall litter, and the condition of residential units [31].

In addition to the two neighborhood measures, raters also recorded the date and time of day they began and completed the rating. Because of the summer/early fall season of this field phase, we also asked raters to record the temperature (Fahrenheit 60–70, 71–80, 81–90, 91–100, and above 100) and whether it was raining during their rating. All ratings were conducted during daylight hours.

### Analyses

#### Scaling

We conducted confirmatory factor analyses to evaluate the factor structure of the seven-item NAS (NAS-7) developed from previous exploratory factor analyses. Prior analyses were based on data in which the AAH NAS and the Krause scales were obtained from different follow-up periods [22,31]. We also examined the Krause five-item scale with confirmatory factor analysis. Model goodness-of-fit indices included Chi-square (> 0.05), Root Mean Square Error of Approximation (RMSEA; < 0.05) and comparative fit index (CFI; >0.90). Scales were also tested for internal consistency (coefficient alpha).

### Comparison by catchment area

Descriptive statistics are reported for items, scales, and interview variables (duration, time between test and retest) as means and standard deviations (SD), or percentages and 95% confidence intervals (95% CI). We examined the scale score distributions, specifically for the potential that there were unusual groupings of scores (e.g., suggesting a pattern of very common ratings) or ceiling effects. We judged there to be ceiling effects if 20% or more of the ratings were at the lowest (best rating) score. We also report descriptive results of items and scales between the two AAH catchment areas (inner city vs. suburbs), and test scale mean differences with a t-test as a test of construct validity.

### Interrater reliability

We examined the agreement between raters on individual items and for the total scores of both measures. For individual items, we used simple Kappa (κ) for dichotomous items, and weighted Kappa for ordered categorical variables with more than two categories as measures of chance-corrected agreement [45,46]. Because Kappa is sensitive to marginal frequencies and prevalence [47], we also computed a prevalence- and bias-adjusted Kappa, the PABAK [48]. We calculated agreement for the overall score using the intraclass correlation coefficient (ICC) using a two-way random effects model [45,49]. We also conducted a sensitivity analysis to examine if interrater reliability varied by time between ratings (≤ 10 days or more than 10 days), and by inner city compared to suburban areas. We classified ICC and κ statistics above 0.75 as excellent agreement and below 0.40 as poor agreement based on recommendations summarized by Fleiss [46].

To explore the potential effects of interviewer characteristics on scoring, we constructed linear models with individual total scale scores as the dependent variable. In the base model, we entered the dichotomous variable for catchment area. In the expanded model, we entered dummy variables representing each interviewer, contrasting our Team Supervisor as the comparison to the other three.

Analyses were conducted using IBM SPSS Statistics version 21 and IBM SPSS AMOS version 21.

### Concurrent validity

We hypothesized that the shorter scales (Krause, NAS short scales) would exhibit concurrent validity by correlating with three key health outcomes: LBFL, the SPPB, and PEF. We used linear regression models for each health outcome. We analyzed these outcomes with three models adding additional adjustments. Model 1 adjusted for age and gender. Model 2 adjusted for age, gender, and area (inner city versus suburbs). Model 3 adjusted for age, gender, area (inner city versus suburbs), and interviewer.

### Mismeasurement analysis

To estimate the effects of measurement error on observed associations, we used regression calibration to calculate the calibrated mean neighborhood conditions. We calculate both the naïve (uncorrected) association of mean (times 1 and 2) neighborhood conditions and the calibrated mean (times 1 and 2) neighborhood conditions with self-rated health. Regression calibration predicts and uses the ‘true’ neighborhood characteristics for each subject to correct effect estimates. Neighborhood conditions are assumed to be measured with random additive error, estimated from test–retest replicated measures, effectively adjusting for test–retest reliability. Using a linear calibration function for replicated data, the calibrated mean for each participant can be calculated as: $X_{i}^{*}={\bar{X}}_{\mathrm{tot}}+\lambda \left({\bar{X}}_{j}-{\bar{X}}_{\mathrm{tot}}\right)$ where ${\bar{X}}_{\mathrm{tot}}$ is the grand mean of all observations, $X_{i}^{*}$ is the mean of the replicate measurements for each participant, and λ is the ICC reliability coefficient [50]. We compared the mean calibrated (regression calibration) odds ratio (OR) that corrects for measurement error to the naïve analysis (uncorrected for measurement error).

## Results

### Descriptive results

A total of 483 ratings and 120 interrater retest pairs were completed during 21 weeks in 2010. On average, ratings took 10 minutes (interquartile range 6 to 9 minutes), and varied among the four raters (mean times 7 to 13 minutes). Rating times also were somewhat shorter for suburban blocks (mean 9 minutes) compared to the inner city (11 minutes). All ratings took place during daylight hours, and most ratings took place in the morning (43.7%) or afternoon (45.7%). Only 2% of ratings occurred when it was raining, and 41.1% occurred when the temperature was 90 degrees F or higher. There were no differences in the score patterns of ratings by time of day or weather.

As expected, the Krause and AAH NAS scale scores were higher, indicating worse neighborhood conditions, in the inner city compared to the suburban neighborhood conditions (Table 1). The mean Krause scale score was 7.5 (SD ± 2.4) in suburban neighborhoods, and 8.6 (SD ± 2.6) in the inner city (p<0.05). The AAH NAS total 18-item score was 7.0 (SD ± 3.2) versus 9.2 (±3.4), the AAH NAS-7 was 3.4 (SD ± 2.8) versus 5.2 (SD ±3.0), and the NAS-5 was 2.1 (SD ± 2.0) versus 3.7 (SD ± 2.2) for suburban and inner city neighborhoods (all p values <0.05). Individual items also demonstrated expected differences, for example detached single family homes were the norm in suburban neighborhoods (98.3% of housing) compared to only about half (53.6% of housing) of inner city neighborhoods. The Krause scale had some ceiling effects, with 23.1% of scores at the minimum value of 5 points and almost 60% of scores from 5 to 8 points. The NAS-7 and NAS-5 scales both had broader distributions than Krause, with only 11.5% and 19.7% of scores at the minimum, respectively.

### Scaling

#### Confirmatory factor analysis (CFA)

**Figure 1:**
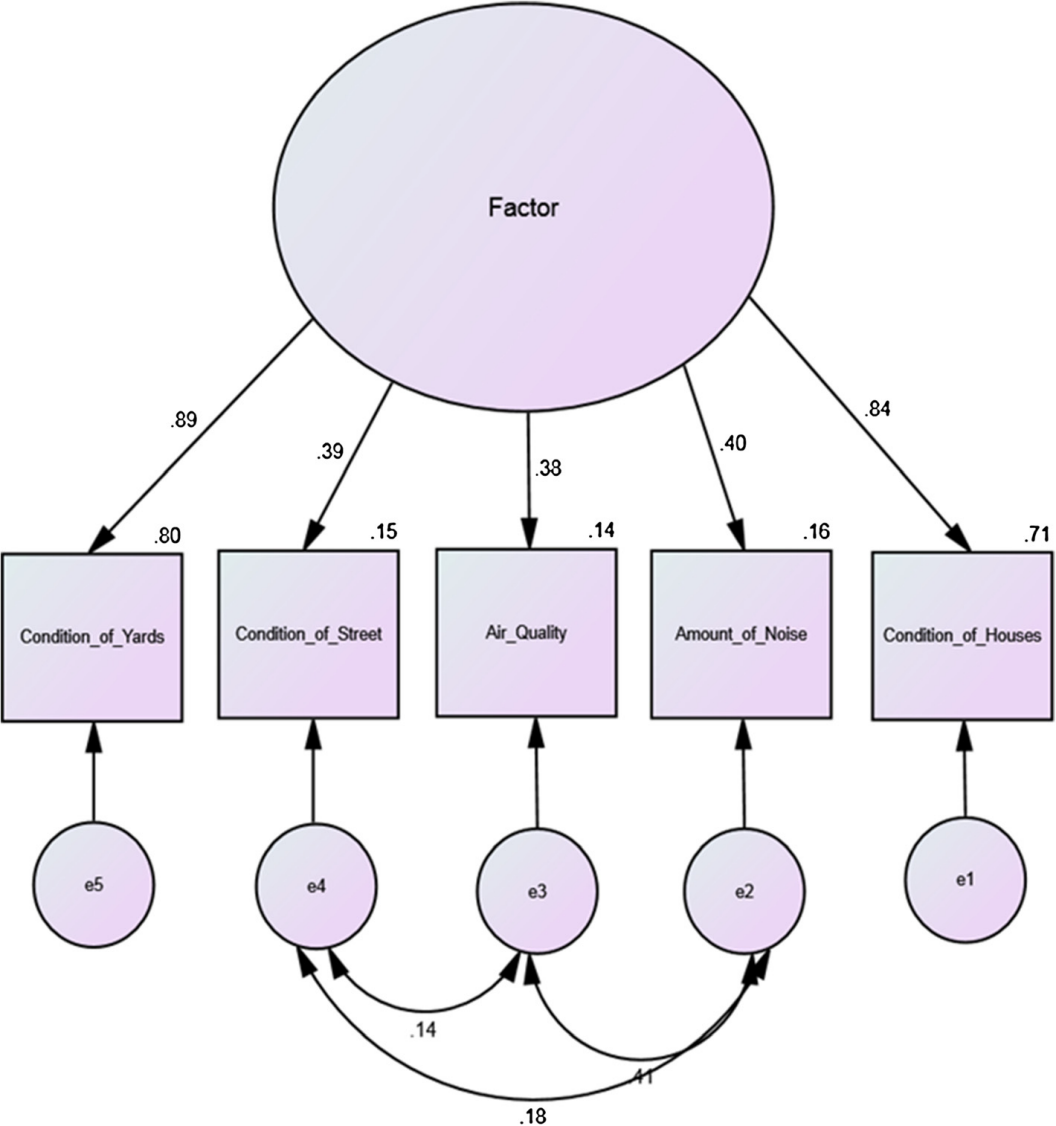
Krause neighborhood assessment scale factor model with standardized estimates.

**Figure 2:**
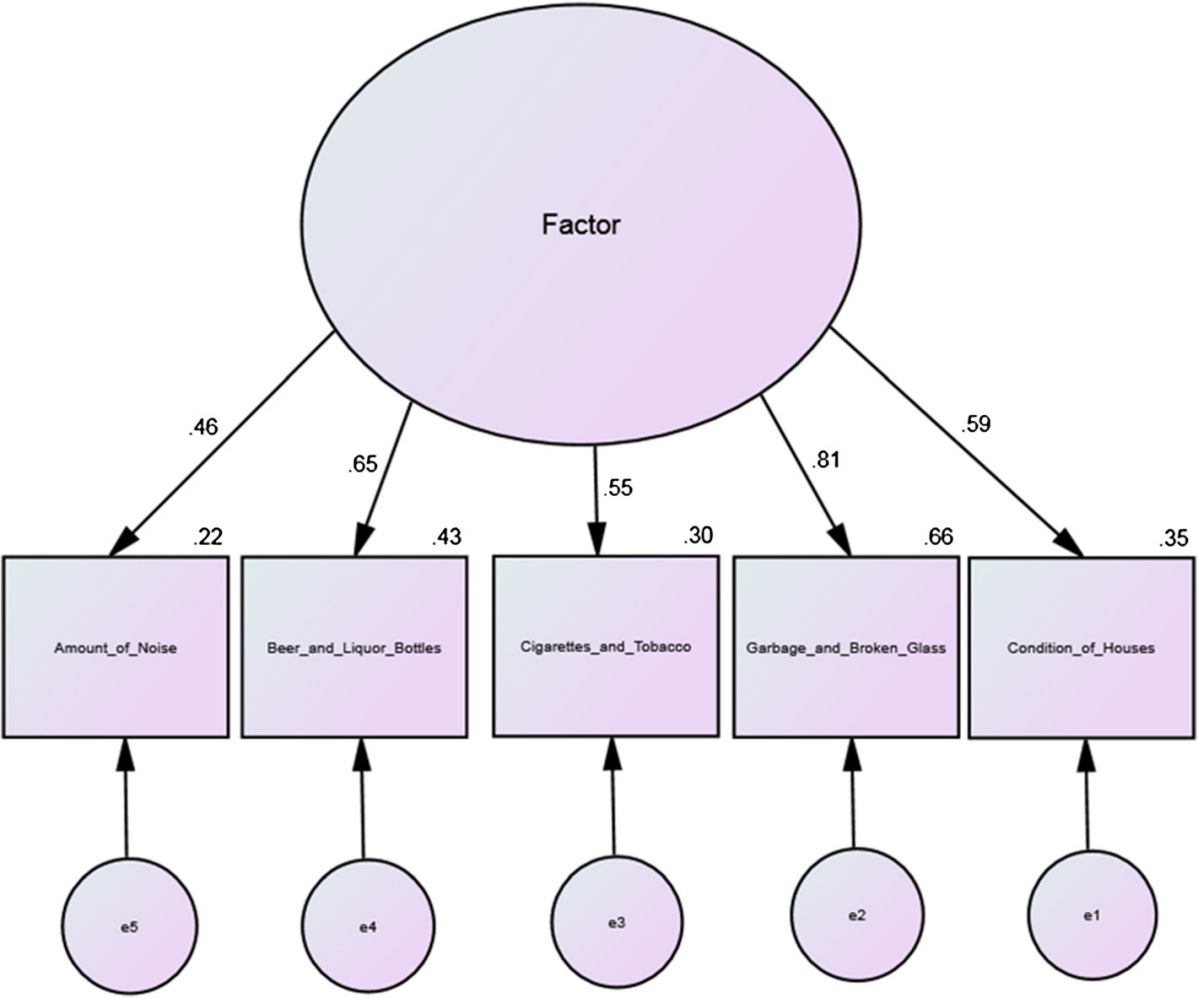
African American Health neighborhood five-item neighborhood assessment scale factor model with standardized estimates.

A previous exploratory factor analysis extracted one factor for the AAH NAS seven-item scale [28]. CFA for the AAH NAS seven-item scale (whether one or two factors) for the present study, however, did not achieve acceptable model fit. An excellent fit was achieved for a CFA of a one factor NAS model with five items (Table 2). The acceptable CFA model included five items describing neighborhood conditions (noise, three litter items, and housing condition), and is shown in Figure 1. CFA for the Krause scale achieved acceptable model (Table 2) fit for the single factor model shown in Figure 2. Coefficient alpha for the NAS-5 was 0.73, for the NAS-7 was 0.74, and for the Kraus was 0.75.

**Table 2:** Confirmatory factor analysis of the Krause scale, and the African American Health Neighborhood Assessment Scales (AAH NAS)

**Scale**	**χ**^**2 **^**(p-value)**	**Root mean square error of approximation**	**Comparative fit index**
**Krause Five-Item scale**			
One factor model	2.6 (0.27)	0.02	0.99
**African American Health Neighborhood Assessment Scales**			
One factor model (5-items)	5.4 (0.37)	0.01	0.99
One factor model (7-items)	310.6 (<0.001)	0.19	0.71
Two factor model (7-items)	92.7 (<0.001)	0.10	0.92

#### Interrater reliability results and interviewer effects

The mean number of days between the first and retest ratings was (12.8±9.8). About half of the retest ratings occurred within two weeks (52%, n=62). Overall, retest reliability was better when the retest time was shorter. The inter-rater ICC was poor for the Krause overall (ICC=0.19: Table 1), but better in the 62 observations retested within ten days (ICC=0.49). The NAS-7 and NAS-5 scales had ICCs of 0.56 and 0.62 overall (Table 1) versus 0.71 and 0.73 when retested within ten days, respectively. In general, item retest К results of both the Krause and AAH NAS were poor (Table 1), and as with the scales, improved with shorter time between ratings (data not shown).

Interviewers demonstrated strikingly different ratings when comparing their raw mean scores (Table 3). For example, for the five-item Krause scale, overall ratings varied among the four interviewers from a mean of 6.2 points to 9.4 points. For the short AAH NAS scales, mean scores among interviewers varied between 2.2 and 5.4 points for the NAS-7 and 1.5 to 3.6 points for the NAS-5. These differences among interviewers persisted when tested in the linear models, adjusting for neighborhood catchment area (Table 4). For the Krause five-item scale, after accounting for neighborhood (scores were 1.2 points higher in the inner city), individual interviewers varied by as much as 3.1 points compared to the Interviewer Supervisor scores. For the brief AAH NAS scores, interviewers also varied by as much as 3.1 points compared to their Supervisor for the NAS-7, and 2.2 points for the NAS-5. As shown in Table 4, the largest differences were based on a single rater (Interviewer # 3), whose scores were lower (indicating better conditions) than the others, and who spent a substantially shorter time on ratings in the inner city than others (Table 3).

**Table 3:** Comparison among four interviewer raters for two neighborhood rating scales

	**Interviewer (mean ± standard deviation) +**				
	**Overall**	1*	**2**	**3**	**4**
**Time** (minutes; n=670)	10 ± 7	7 ± 3 (n=112)	10 ± 9 (n=187)	7 ± 5 (n=184)	13 ± 5 (n=187)
Inner City (n=287)	11 ± 7	9± 4	12 ± 9	6 ± 3	15 ± 7
Suburbs (n=383)	9 ± 7	6± 3	9 ± 10	8 ± 6	13 ± 5
**Krause scale** (n=688)	8.0 ± 2.6	9.4 ± 2.3 (n=111)	7.4 ± 2.4 (n=205)	6.2 ± 1.3 (n=185)	9.4 ± 2.5 (n=187)
Inner City (n=293)	8.6 ± 2.5	9.5 ± 2.3	8.60 ± 2.5	6.70 ± 1.4	10.1 ± 2.5
Suburbs (n=395)	7.5 ± 2.5	9.3 ± 2.3	6.40 ± 1.7	5.9 ± 1.1	9.0 ± 2.5
**AAH 7-item scale** (n=683)	4.2 ± 3.0	5.4 ± 2.9 (n=112)	4.2 ± 2.6 (n=204)	2.2 ± 2.1 (n=182)	5.5 ± 3.3 (n=185)
Inner City (n=291)	5.2 ± 3.1	6.4 ± 2.8	5.2 ± 2.6	2.9 ± 2.0	6.8 ± 3.1
Suburbs (n=392)	3.5 ± 2.8	4.9 ± 2.8	3.3 ± 2.3	1.6 ± 1.9	4.6 ± 3.1
**AAH 5-item scale** (n=683)	2.8 ± 2.3	3.6 ± 2.4 (n=112)	2.8 ± 2.0 (n=204)	1.5 ± 1.6 (n=182)	3.5 ± 2.5 (n=185)
Inner City (n=291)	3.7 ± 2.3	4.6 ± 2.4	3.7 ± 2.0	2.3 ± 1.6	4.5 ± 2.4
Suburbs (n=392)	2.1 ±2.0	3.1 ±2.2	2.1 ± 1.6	0.9 ± 1.3	2.7 ±2.2
**AAH 18-item scale** (n=681)	8.0 ± 3.4	9.1 ± 3.1 (n=112)	7.9 ± 2.9 (n=203)	5.7 ± 2.4 (n=182)	9.6 ± 3.7 (n=185)
Inner City (n=291)	9.1 ± 3.4	10.1 ± 3.2	9.1 ± 2.9	6.8 ± 2.4	10.9 ± 3.6
Suburbs (n=390)	7.1 ± 3.2	8.6 ± 3.0	6.8 ± 2.5	4.8 ± 2.0	8.6 ±3.4

+Includes primary baseline and retest ratings.

*Interviewer 1 was the field supervisor and was assigned relatively fewer ratings.

**Table 4:** Interviewer effects on neighborhood rating scale scores (n=569†)

	**Krause Five-Item Scale+**		**African American Health Neighborhood Assessment Scales**			
			**(AAH NAS) +**			
**Variable (total ratings)**	Β coefficient^*^	**p-value**	Β coefficient^*^		**p-value**	
			**7-item**	**5-item**	**7-item**	**5-item**
*Constant*	8.86	<0.001	4.53	2.93	<0.001	<0.001
Catchment area Inner City vs. Suburbs	1.23	<0.001	1.84	1.67	<0.001	<0.001
**Interviewers** (vs. Interviewer 1 [n=96]) #						
Interviewer 2 (n=169)	−1.96	<0.001	−1.14	−0.87	<0.001	<0.001
Interviewer 3 (n=152)	−3.12	<0.001	−3.13	−2.16	<0.001	<0.001
Interviewer 4 (n=152)	0.03	0.924	0.22	−0.15	0.523	0.565
**R**^**2 **^**for this model**	0.32		0.28 (7 item)	0.27 (5-item)		

†Observations are all the original (first) rating. Excludes retested duplicated observations.

^#^Interviewer 1 was the field supervisor for the AAH neighborhood rating phase.

^+^Krause scale scores range from 5–20 (Range for this test was 5–16); AAH 7-item Scale scores range from 0–17 (Range for this test was 0–13) and for the AAH 5-item Scale scores range from 0–11 (Range for this test was 0–9).

^*^Unstandardized beta coefficients.

In general, the relationship between both rating scales and our key health outcomes were in the direction predicted, although the magnitude was not uniformly statistically significant (Table 5). For example, higher (worse) lower body function was positively correlated to higher rating scales (lower neighborhood quality), although these were not statistically significant except for the minimally adjusted model for both short AAH NAS. The strongest statistical relationship for both scales was for PEF. The strength of the relationship (unstandardized beta) was strongest for the majority of outcomes models for the Krause and AAH NAS-7 when adjusted by interviewer (Model 3), suggesting interviewer variability partly explained the relationship. The AAH NAS-5 did not follow the pattern and interviewer adjusted models (Model 3) were virtually identical to Model 2 (Table 5).

**Table 5:** Concurrent validity of neighborhood rating scale scores on key health outcomes

**Krause Five-Item Scale**			
**Linear models**^**1,2**^	**Model 1**	**Model 2**	**Model 3**
Lower body function^3^ (scores 0–5) n=539	0.038 (0.03)	0.027 (0.03)	0.025 (0.04)
Short physical performance battery^4^ (scores 0–12) n=488	−0.098 (0.04)*	−0.077 (0.04)	−0.88 (0.5)
Peak expiratory flow (PEF)^4^ (100–850) n=441	−4.961 (1.85)**	−4.453 (1.92)*	−6.283 (2.24)**
**African American Health Neighborhood Seven-Item Assessment Scale (7-item AAH NAS)**			
**Linear models**^**1,2**^	**Model 1**	**Model 2**	**Model 3**
Lower body function (scores 0–5) n=535	0.063 (0.03)*	0.053 (0.03)	0.056 (0.03)
Short physical performance battery (scores 0–12) n=487	−0.093 (0.04)**	−0.074 (0.04)*	−0.074 (0.04)
Peak expiratory flow (PEF)^4^ (100–850) n=440	−5.567 (1.57)***	−5.243 (1.65)**	−5.511 (1.85)**
**African American Health Neighborhood Five-Item Assessment Scale (5-item AAH NAS)**			
Lower body function (scores 0–5) n=535	0.089 (0.04)*	0.075 (0.04)*	0.075 (0.04)
Short physical performance battery (scores 0–12) n=487	−0.127 (0.05)**	−0.098 (0.05)	−0.095 (0.06)
Peak expiratory flow (PEF)^4^ (100–850) n=440	−6.377 (2.11)**	−5.868 (2.27)*	−5.647 (2.48)*

^1^Unstandardized beta coefficient (± standard error). Model 1 adjusted for age and gender. Model 2 adjusted for age, gender, and area (inner city versus suburbs). Model 3 adjusted for age, gender, area (inner city versus suburbs), and interviewer (#1 versus 2, 3, & 4).

^2^* p <0.05, ** p<0.01, *** p<0.001.

^3^Lower body function sums difficulties, and correlations are positive with the same direction of poorer neighborhood ratings.

^4^Short Physical Performance Battery and Peak Expiratory Flow both yield higher values for better health and correlations are negative with the reverse direction of the rating scales.

#### Mismeasurement effects

Of all 582 participants at wave 10, 35.6% rated their health as fair or poor. Both the naïve (uncorrected) and regression-calibrated results showed that participants were more likely to report being in fair or poor health when living in neighborhoods with worse observed conditions, although the confidence interval included the null value in some instances. In general, the naïve estimates for all scales were biased towards the null compared with measurement-error corrected estimates (Table 6).

**Table 6:** Odds ratios* for naïve (unadjusted) and measurement error corrected associated between neighborhood scale and fair-poor (vs. good, very good, or excellent) self-rated health status, adjusted for age and sex

	**Krause scale**	**7-item AAH NAS+**	**5-item AAH NAS+**	**18 -item AAH NAS+**
	**Odds ratios (95% confidence interval)**			
**Naïve estimates**	1.04 (0.98 – 1.12)	1.06 (1.01 – 1.13)	1.10 (1.01 – 1.18)	1.05 (1.00 – 1.10)
**Error corrected**	1.32 (0.18 – 9.60)	1.16 (0.99 – 1.36)	1.22 (1.01 – 1.46)	1.11 (0.97 – 1.28)

*Estimates are per point on a scale.

+ Neighborhood Assessment Scale.

## Discussion

The Krause and AAH NAS scales demonstrated good construct ability, with higher scores (worse conditions) for inner city compared to suburban St. Louis neighborhoods. The AAH NAS-5 had especially good discrimination, with a mean of 3.7 (SD ±2.2) for inner city compared to 2.1 (±2.0) for suburban neighborhoods. Both short AAH scales were less skewed and showed less ceiling effect than the Krause, and only 19.7% of scores of the NAS-5 were at the minimum (worse) rating of 0 points. We previously found the Krause rating scale to have a problem with a narrow range of scores [22], and our enhanced training did seem to yield a broader range of scores. Concurrent validity of key physical and lung functions also showed promise, and were especially strong for an association with Peak Expiratory Flow (PEF). In our earlier tests of the Krause and AAH NAS measures at different waves of data collection, we also compared the results from our interviewers to global ratings from the participants and found that Inner City residents rated their neighborhoods as “worse” compared to suburban residents, and that their global ratings had strong linear trends with worse interviewer scores across participants’ rating categories [22,31].

While a one-factor solution was again observed for the Krause, the AAH NAS-7 resulted in two factors, one measuring neighborhood conditions and one measuring neighborhood activities, and did not provide an acceptable fit in the CFA. The NAS-5 CFA had an excellent fit to the data (CFI and RMSEA). The NAS-5 also demonstrated the best retest ICC (0.62). However, the NAS-7 continued to demonstrate concurrent validity.

Despite generally positive results of concurrent and construct validity, these rating measures had relatively weak interrater reliability (especially at the individual item level). Because individual items may have had skewed response ratings (e.g., very few abandoned cars were observed and few ratings suggested smells were a problem), we recommend caution interpreting the raw item Kappa values [47,48]. Our enhanced training and field methods designed specifically to increase protocol consistency failed to fully eradicate interviewer variability. Among four highly experienced interviewers, we noted that one of them produced scales with much lower (better) scores for the inner city neighborhoods. In separate analyses, both item and scale reliability improved when this individual’s ratings were removed (data not shown). We and others have reported higher interrater reliability for various neighborhood rating measures [22,26,50,51], however the reliability effects of varied interviewer training and the effect of score compression of ratings are still unclear. Based on our prior experience with low variability among Krause Scale scores [22], we specifically used a protocol that sought to increase the overall quality of raters and their training and to decrease the potential for variability of ratings across raters. These improvements did not improve interrater reliability for the Krause Scale. In other real world community research, there could be one or more data collectors who systematically rate observations differently, and perhaps the more important lesson learned is what effect this has on the analysis posed. In our test, while the mismeasurement attenuated the magnitude of relationships between neighborhood conditions and self-rated health, it did not obscure them.

Our research and these results have several limitations. First, our population was chosen to investigate urban African American health and aging, and generalizability to multiracial populations, other cities, and rural settings is unknown. Second, our selection of scales and neighborhood items has some distinctive characteristics, and our experience with training and interviewer variability may be unique. St. Louis has been the site of other research on effects of neighborhood conditions with a different rating system intended to investigate how conditions affect walkability [23,24] resulting in greater overall retest reliability for fairly objective rating items (e.g., presence of specific business types), but lower ICC for more subjective items (e.g., parking difficulties, walking difficulty due to hills). In a recent report of a different observational scale of neighborhood characteristics, McDonell and Waters [26] described retest reliability that also was relatively modest (ICC = 0.54). However, other rating scales reports have sparse information regarding potential measurement errors.

## Conclusions

With few comparison data in the published literature about training protocols and tests of the validity and reliability for other neighborhood observer rating systems, we conclude that such measures may include an inherent degree of variability. Our findings that mismeasurement can attenuate statistical relationships suggests that the “signal” associated with neighborhood effects may be quite strong. However, we urge additional examination of the measurement properties of all environmental rating methods and a thorough discussion of field protocols and rater training. Comparison among these experiences and measurement tests could yield an overall more robust science for this important research.

## Competing interests

The authors declare that they have no competing interests.

## Authors’ contributions

EMA conceived the study; planned and trained staff; and supervised the field data collection for the neighborhood rating; contributed to the analyses plans; and was the lead author writing and revising the manuscript. TKM performed the psychometric analyses and contributed to the writing and revision of the manuscript. MS designed the GIS methods; performed the mismeasurement analysis; and contributed to theory and analysis methods, writing, and revision of the manuscript. FDW, JPM, and DKM contributed to design of the overall project, the present analyses, and the review and revision of the manuscript. All authors read and approved the final manuscript.

## Pre-publication history

The pre-publication history for this paper can be accessed here:

http://www.biomedcentral.com/1471-2458/13/1024/prepub
